# A novel missense mutation in the gene encoding major intrinsic protein (*MIP*) in a Giant panda with unilateral cataract formation

**DOI:** 10.1186/s12864-021-07386-8

**Published:** 2021-02-02

**Authors:** Chao Bai, Yuyan You, Xuefeng Liu, Maohua Xia, Wei Wang, Ting Jia, Tianchun Pu, Yan Lu, Chenglin Zhang, Xiaoguang Li, Yanqiang Yin, Liqin Wang, Jun Zhou, Lili Niu

**Affiliations:** 1 Beijing Key Laboratory of Captive Wildlife Technologies, Beijing Zoo, Beijing, China; 2 Beijing Zoo, Beijing, China; 3 Chongqing Zoo, Chongqing, China; 4 Chengdu Zoo, Chengdu, China

**Keywords:** Cataracts, Giant panda, Major intrinsic protein (MIP)

## Abstract

**Background:**

Cataracts are defects of the lens that cause progressive visual impairment and ultimately blindness in many vertebrate species. Most cataracts are age-related, but up to one third have an underlying genetic cause. Cataracts are common in captive zoo animals, but it is often unclear whether these are congenital or acquired (age-related) lesions.

**Results:**

Here we used a functional candidate gene screening approach to identify mutations associated with cataracts in a captive giant panda (*Ailuropoda melanoleuca*). We screened 11 genes often associated with human cataracts and identified a novel missense mutation (c.686G > A) in the *MIP* gene encoding major intrinsic protein. This is expressed in the lens and normally accumulates in the plasma membrane of lens fiber cells, where it plays an important role in fluid transport and cell adhesion. The mutation causes the replacement of serine with asparagine (p.S229N) in the C-terminal tail of the protein, and modeling predicts that the mutation induces conformational changes that may interfere with lens permeability and cell–cell interactions.

**Conclusion:**

The c.686G > A mutation was found in a captive giant panda with a unilateral cataract but not in 18 controls from diverse regions in China, suggesting it is most likely a genuine disease-associated mutation rather than a single-nucleotide polymorphism. The mutation could therefore serve as a new genetic marker to predict the risk of congenital cataracts in captive giant pandas.

**Supplementary Information:**

The online version contains supplementary material available at 10.1186/s12864-021-07386-8.

## Background

Cataracts are heterogeneous and multifactorial eye lesions in which the lens becomes opaque due to the accumulation of pigments and protein aggregates induced by progressive oxidative damage [1, 2]. Many cataracts are acquired, age-related lesions but approximately one third of cases have a significant genetic component, and most of these congenital forms are transmitted as autosomal dominant traits with strong penetrance but varying degrees of expressivity [3]. Although the pathogenesis of cataracts often has a genetic component, the etiology is complex because progression is also influenced by nutrition, metabolism and the environment. Cataract formation is therefore the long-term consequence of multiple intrinsic and external factors. For example, epidemiological studies have shown that human cataract development is promoted by ultraviolet radiation, diabetes, hypertension, cardiovascular disease, body trauma, and excess drinking and smoking [4, 5].

Whereas some congenital cataracts are caused by the disruption of eye development, others reflect the presence of mutations in genes required for normal lens function [2]. For example, in humans, underlying mutations have been detected in genes encoding transcription factors that regulate lens activity, such as *PITX3* [6] and *HSF4* [7], and in genes encoding lens cytoskeletal proteins, such as *BFSP2* [8, 9]. Several mutations have been traced to genes encoding crystallin proteins, which normally remain soluble and confer transparency, including α-crystallins [10], β-crystallins [11–13], and γ-crystallins [14, 15]. Another major category of cataract-promoting mutations affect genes encoding lens membrane channels or gap junction proteins, such as connexin 46 (*GJA3*) [16] and connexin 50 (*GJA8*) [17]. One of the most important membrane channels in the context of cataract formation is the lens major intrinsic protein (MIP), also known as aquaporin 0 (AQP0) [18].

MIP/AQP0 is an integral membrane protein (28 kDa, 263 amino acids) with six transmembrane domains, which assembles into a tetramer containing four independent water channels [19, 20]. It is expressed at high levels in lens fiber cells and constitutes ~ 45% of the total membrane protein [21]. Its main function is the transport of water and small, neutral solutes [22–24], but it is also required for the adhesion of lens fiber cells via interactions with crystallins and connexin 50 [25–27]. At least 19 mutations in the human *MIP* gene (Table 1) have been linked to autosomal dominant cataracts with diverse phenotypes, reflecting the multi-domain and multi-functional nature of the protein [28–45]. In many cases, these mutations reduce the abundance of MIP and/or prevent normal trafficking to the plasma membrane, thus inhibiting water and solute transport as well as cell–cell interactions [23, 37, 46]. Mutations in the mouse *Mip* gene have also been linked to genetic cataracts, such as Fraser (*Cat*^*Fr*^), lens opacity (*lop*), *Hfi*, *Tohm* and *Nat* [47–50]. The loss of water permeability in *mip*-deficient mice [20] can be rescued by the expression of AQP1 [51]. However, this does not restore the ordered packing of the lens fiber cells and still results in the formation of cataracts, confirming that MIP has unique functions in the lens that are not complemented by other aquaporins [51].

**Table 1:** Known mutations in the human *MIP* gene compared to the novel mutation in the panda *MIP* gene

Exon/intron	DNA Change	Coding Change	Inheritance	Origin	Phenotype	Species	Reference
Exon 1 (p1–120)	c.2T > C	p.M1T	AD	China	Initiation codon mutation	Human	Xiao et al., 2011 [35]
	c.97C > T	p.R33C	AD	China	Missense	Human	Gu et al., 2007 [30]
	c.319G > A	p.V107I	AD	China	Missense	Human	Wang et al., 2010 [33]
	c.337C > T	p.R113*	AD	China	Nonsense mutation	Human	Yu et al., 2014 [40]
Exon 2 (p121–175)	c.401C > G	p.E134G	AD	UK	Missense	Human	Berry et al., 2000 [28]
	c. 413C > G	p.T138R	AD	UK	Missense mutation	Human	Berry et al., 2000 [28]
	c.448G > C	p.D150H	AD	China	Missense	Human	Shentu et al., 2015 [41]
	c.494G > A	p.G165D	AD	South Indian	Missense mutation	Human	Senthilet al., 2013
	c.508dupC	p.L170fs	AD	China	Missense	Human	Qin et al., 2016 [43]
Exon 3 (p176–202)	c.530A > G	P.Y177C	AD	China	Missense	Human	Yang et al., 2011 [36]
	c.559C > T	p.R187C	AD	China	Missense	Human	Wang et al., 2011 [34]
Intron3	IVS3 − 1G > A		AD	China	Splice-acceptor mutation	Human	Jiang et al., 2009 [32]
	(c.606 + 1G > A)		AD	China	Splice-donor mutation	Human	Zeng et al., 2013 [38]
Exon 4 (p230–263)	c.634G > C	p.G212R	AD	China	Missense	Human	Jiang et al., 2017 [44]
	c.638delG	p.G213fs	AD	US	Frame shift mutation	Human	Geyer et al., 2006 [29]
	c.644G > A	p.G215D	AD	China	Missense	Human	Ding et al., 2014 [39]
	c.657C > G	p.Y219*	AD	China	Nonsense mutation	Human	Song et al., 2015 [42]
	c.682_683delAA	p.K228fs	AD	China	Frame shift mutation	Human	Long et al., 2018 [45]
	c.702G > A	p.R233K	AD	China	Missense mutation	Human	Lin et al., 2007 [31]
***Exon 4***	***G > A***	***p.S229N***		***China***	***Missense***	***Panda***	***This study***

Although mutations affecting MIP have been shown to cause cataracts in humans and mice, analogous mutations have not been reported in the giant panda (*Ailuropoda melanoleuca*). These animals also tend to develop cataracts in captivity because they live much longer than their counterparts in the wild, and they may therefore be exposed to additional risk factors. This phenomenon has been observed in companion animals: for example, cataract development in dogs is often associated with diabetes, obesity, prolonged use of corticosteroid, excessive exposure to sunlight, or previous eye injury/inflammation [52, 53]. It is therefore unclear whether cataracts in captive pandas are age-related acquired or congenital lesions due to the absence of suitable genetic markers [54]. Here we used a functional candidate gene screening approach to test 11 known cataract-associated genes in giant panda specimens with and without cataracts. We identified and characterized a novel missense mutation in the *MIP* gene of a female panda diagnosed with progressive cortical punctate cataracts. The mutation was not present in 18 healthy controls. The identification of this mutation will help to determine the prevalence of congenital cataracts in pandas, and will provide a new diagnostic tool for cataract risk assessment in the zoo environment.

## Results

### Clinical findings

**Fig. 1:**
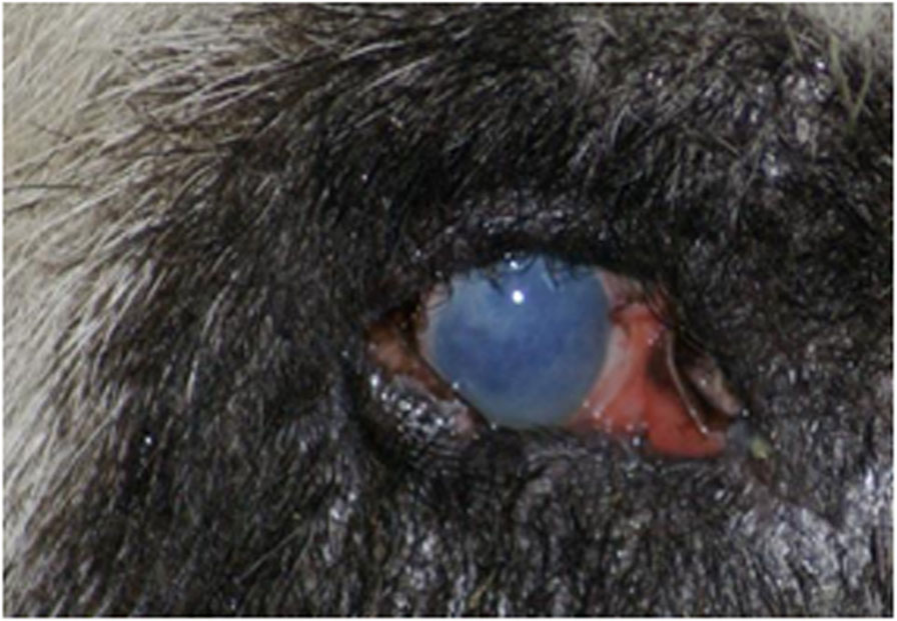
The right eye of Jini, a female giant panda with a unilateral senile cataract

The proband in this study was Jini, a giant panda born in 1993. Routine physical examination were carried out every month for captive pandas, including eye, mouth, nose and physical appearance examination, abdominal palpation, etc. Blood were collected once a month for detection of various physiological and biochemical indicators. Risk factors that affect or cause cataract formation such as injury, diabetes or other factors can be well excluded through examination. Jini’s mild cataract symptoms were first observed in 2013, and in 2017 the lesion was diagnosed as a unilateral senile (age-related) cataract following a professional examination by an ophthalmologist (Fig. 1). However, in the absence of genetic data it was not possible to confirm whether the cataract was acquired or congenital. The ophthalmologist’s diagnosis represented the transition from initial cataract formation to the immature stage of a cortical cataract, and accordingly the pupil area was not occluded and there was only slight visual impairment. In this condition, the cortex absorbs water and swells, the lens volume increases, and the anterior chamber becomes shallow, accompanied by mild secondary glaucoma. Jini’s case records indicated no history of eye trauma or other diseases. We therefore selected Jini for genetic analysis in order to screen for genetic markers that can be used to differentiate between congenital and acquired cataracts. We selected 18 controls without cataracts, including all traceable relatives of Jini and unrelated controls from diverse geographical locations within China (Table 2). This was necessary to distinguish disease-associated mutations from irrelevant single-nucleotide polymorphisms (SNPs).

**Table 2:** Characteristics of the proband and control specimens

		Birth year	Sex	Status	Origin	Mutation	Cataracts	Comments
1	S1 (proband)	1993	Female	Alive	Beijing	+	+	
2	S2	1996	Female	Dead	Beijing	–	–	
3	S3	1986	Female	Dead	Beijing	–	–	
4	S4	1992	Male	Alive	Beijing	–	–	Grandfather of Jini’s offspring
5	S5	1999	Male	Alive	Beijing	–	–	
6	S6	2013	Male	Alive	Beijing	–	–	
7	S7	1998	Male	Dead	Beijing	–	–	
8	S8	1986	Male	Dead	Beijing	–	–	Jini’s father
9	S9			Alive	Beijing	–	–	
10	S10	1982	Female	Alive	Baoxing	–	–	
11	S11	1999	Male	Alive	Baoxing	–	–	Father of Jini’s offspring
12	S12	2007	Male	Alive	Yaan	–	–	
13	S13	2009	Male	Alive	Yaan	–	–	
14	S14	2009	Female	Alive	Yaan	–	–	
15	S15	2011	Female	Alive	Yaan	–	–	
16	S16	1998	Female	Alive	Wolong	–	–	
17	S17		Female	Dead	Wolong	–	–	
18	S18	2003	Female	Alive	Wolong	–	–	
19	S19	1989	Female	Dead	Chengdu	–	–	

### Mutation detection

**Fig. 2:**
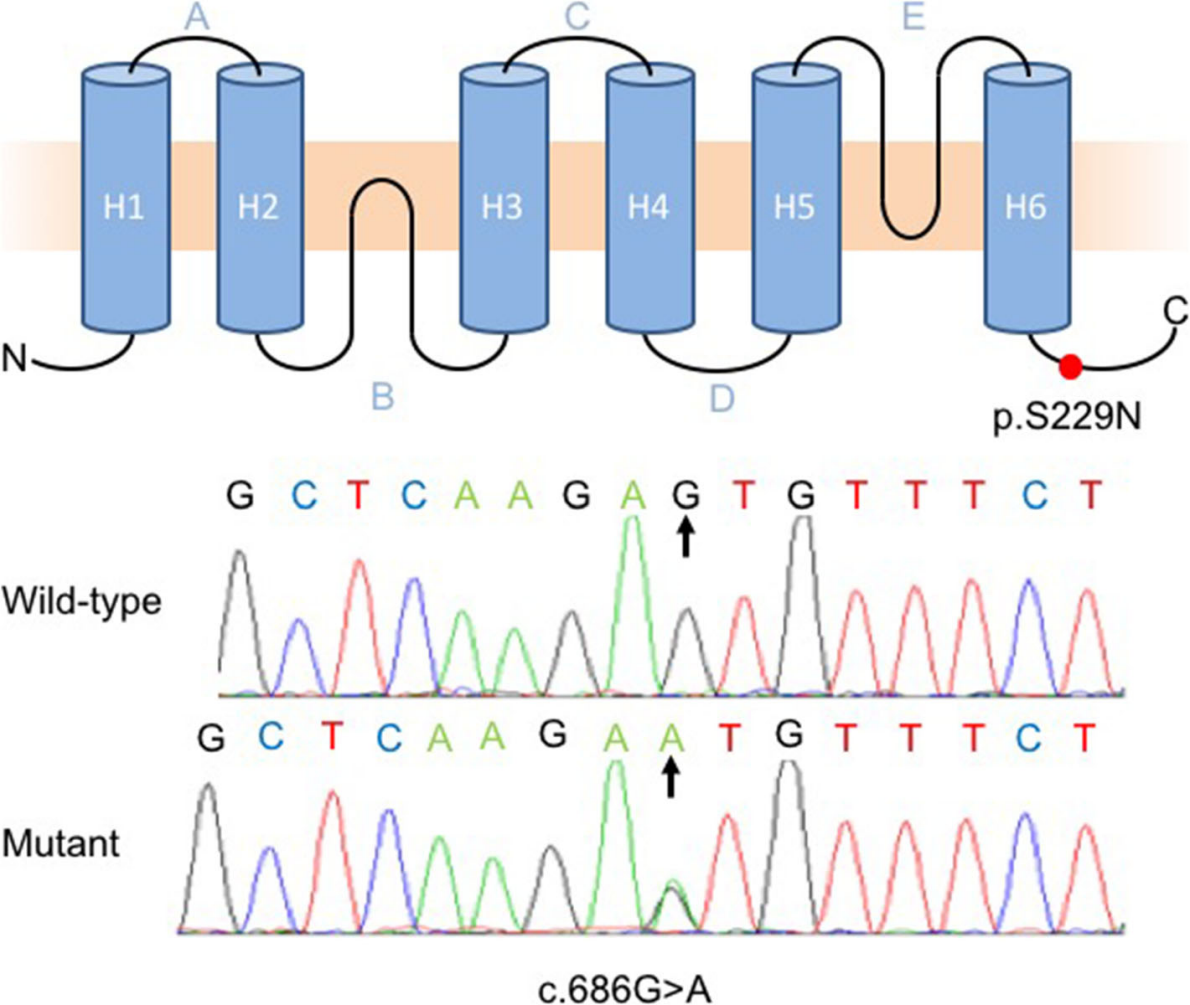
Characterization of the mutation in the *MIP* gene of Jini. (**a**) Extended structure of MIP, showing the six transmembrane domains (H1–H6), extracellular loops (A/C/E), intracellular loops (B/D), the intracellular N-terminal portion, and the intracellular C-terminal tail, the latter containing the mutation site (red dot). (**b**) Sequence trace of the 16-bp region spanning the mutation site, comparing the 18 controls (top) and Jini (bottom), revealing the heterozygous mutation (**c**.686G > A)

Genomic DNA extracted from Jini and the 18 healthy controls was screened for mutations in 11 candidate genes often associated with cataracts in humans (*CRYAB*, *CRYBA1*, *CRYBB1*, *CRYGC*, *HSPB6*, *HSPB7*, *HSPB9*, *GJA3*, *AQP3*, *MIP* and *HSF4*). This revealed a novel missense mutation in exon 4 of the *MIP* gene (c.686G > A) in Jini but in none of the controls. The transition causes the replacement of a serine residue with arginine at position 229 (p.S229N) in the intracellular C-terminal tail of the protein (Fig. 2). We found that Jini is heterozygous for this mutation.

### Structural analysis

**Fig. 3:**
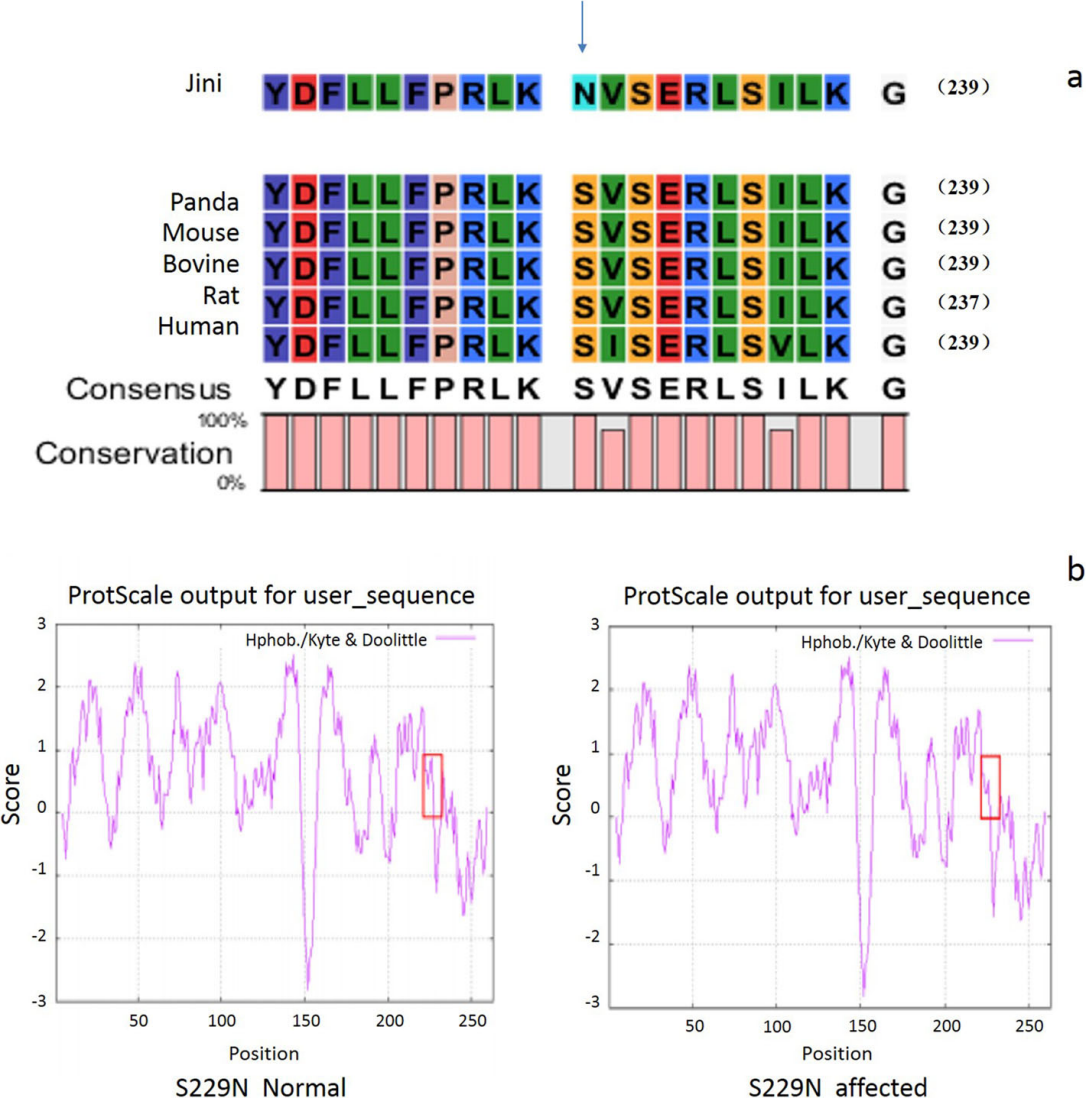
The p.S229N mutation within the intracellular C-terminal domain of MIP affects protein hydrophobicity. **a** Multiple alignment of a highly-conserved sequence of 21 amino acids in five orthologs of MIP (panda, mouse, bovine, rat and human) showing that the panda p.S229N substitution affects a serine residue conserved across all species. **b** ProtScale analysis of the human protein with the equivalent mutation (p.S229N) confirming a decrease in overall hydrophobicity

The amino acid sequences of human, bovine, rat, mouse and panda MIP were aligned, revealing broad conservation throughout the sequence and almost complete conservation in the 10 residues either side of the mutation site, with the only substitutions involving chemically near-identical isoleucine and valine residues (Fig. 3a). The replacement of serine with asparagine within this region therefore swaps a small polar side chain for another that is chemically similar but physically larger, with the potential to form additional hydrogen bonds. ProtScale analysis confirmed that the corresponding mutation in the human MIP protein (p.S229N) would cause a decrease in overall hydrophobicity (Fig. 3b). The potential damaging effect of p.S229N was also predicted by PROVEAN analysis, which generated a score of − 0.805, indicating a neutral mutation.

**Fig. 4:**
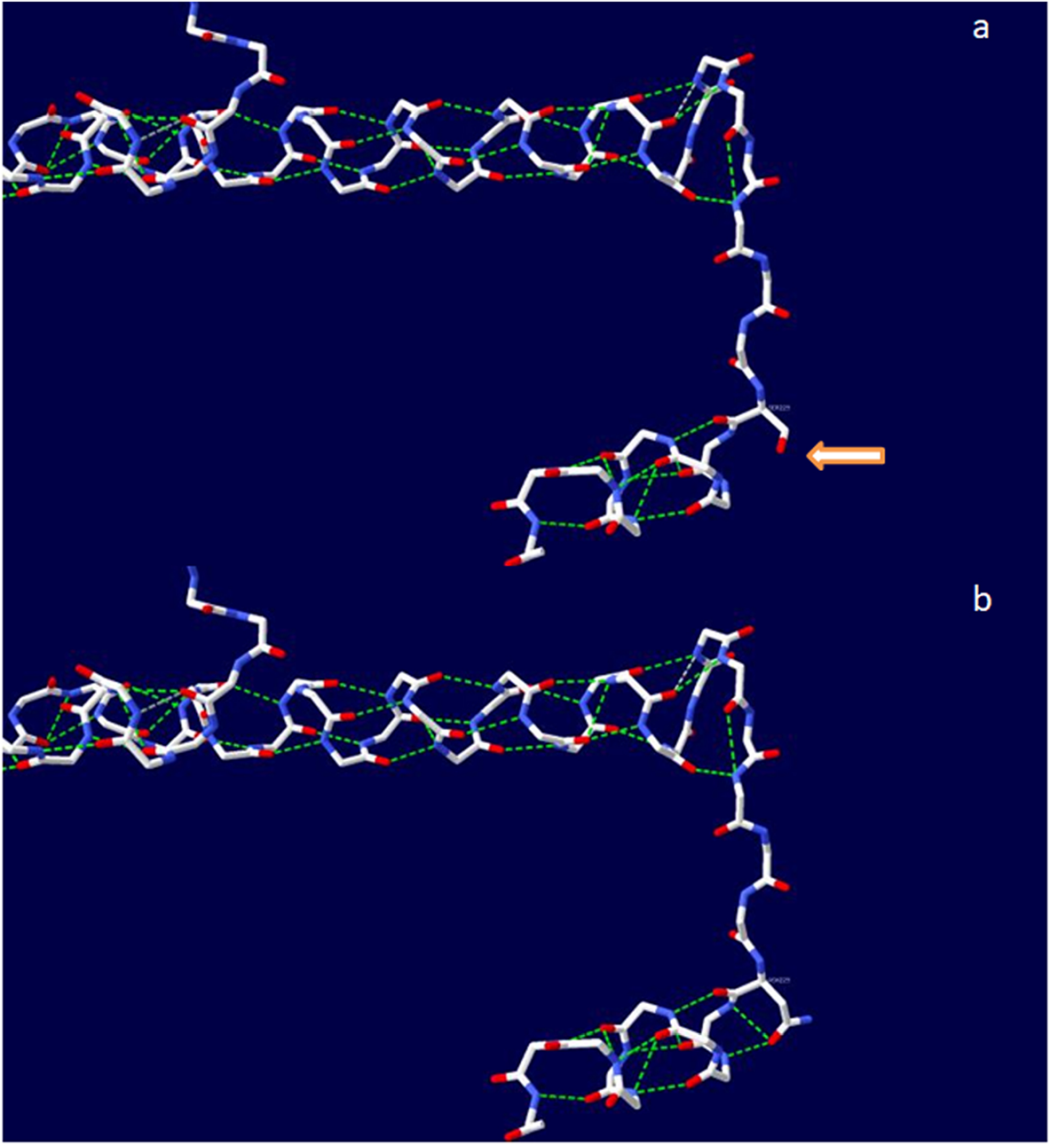
The path of the MIP polypeptide backbone predicted using SWISS-MODEL. **a** Model of wild-type human MIP. **b** Model of the p.S229N mutant. The arrows indicate the difference in intramolecular interactions between wild-type MIP and the p.S229N mutant, with the latter able to form two new hydrogen bonds (shown as broken green lines)

**Fig. 5:**
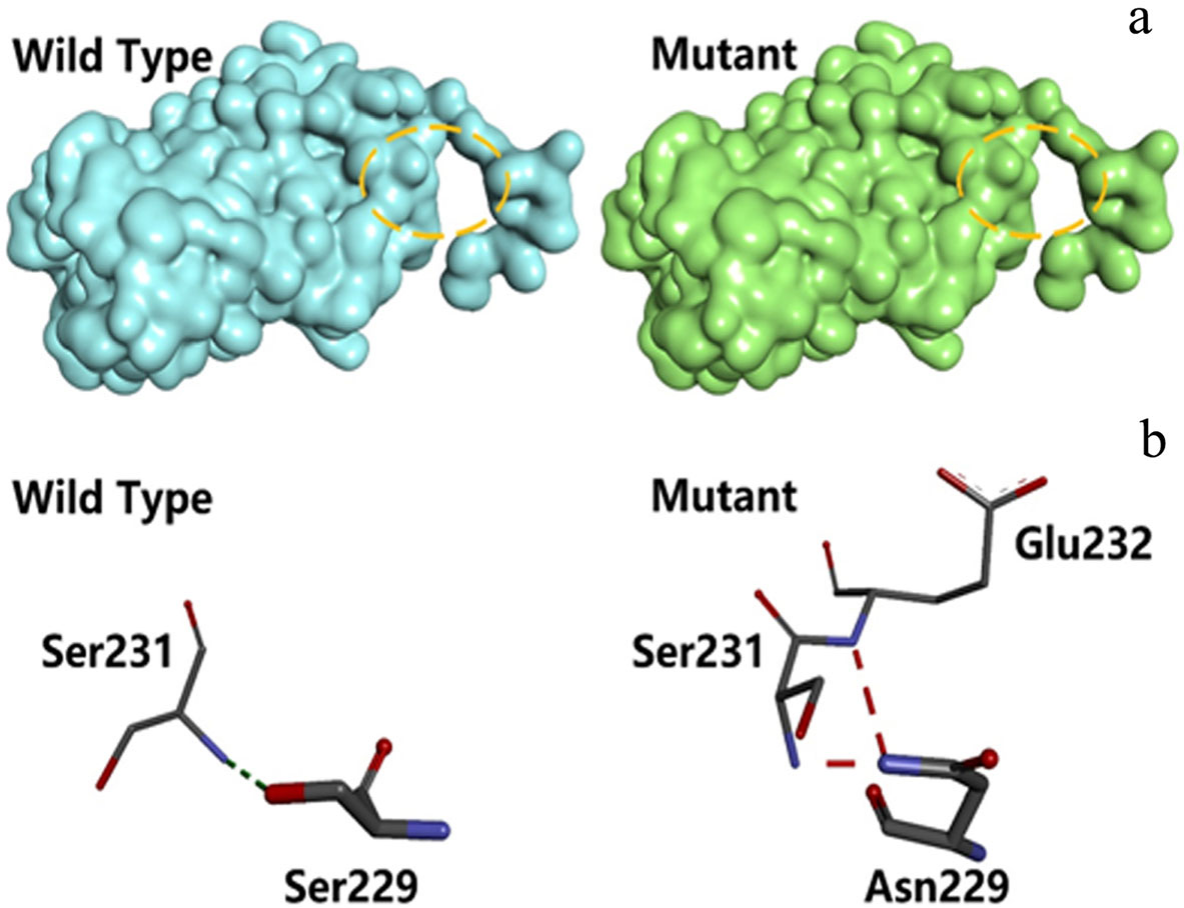
Predicted changes in the tertiary structure of giant panda MIP caused by the missense mutation p.S229N. **a** Changes in surface structure visualized using Discovery Studio Visualizer. The yellow circle reveals a subtle but distinct protrusion in the mutant protein caused by the bulkier side chain of asparagine compared to serine. **b** Interactions between amino acid side chains predicted using THREADER and Modeller. The p.S229N mutant forms two new hydrogen bonds

Structural predictions in SWISS-MODEL showed that the path of the MIP polypeptide backbone is altered by the mutation due to the addition of two hydrogen bonds, increasing the attraction between residue 229 and nearby amino acids (Fig. 4). Following sequence alignment using Clustal X v2.0, the impact of the mutation on protein structure was predicted using Modeller v9.22 with the sheep (*Ovis aries*) MIP (PDB: 2B6O) as a template, revealing discrete changes on the protein surface (Fig. 5a). As shown in Fig. 5b, Ser229 in wild-type MIP forms a hydrogen bond with Ser231, whereas Asn229 in the mutant forms two weak hydrogen bonds with Ser231 and Glu232. These subtle changes in the surface properties and intramolecular interactions are likely to influence the behavior of the C-terminal tail of panda MIP and thus promote the formation of cataracts.

## Discussion

Cataracts can be caused by mutations that affect the activity of several groups of lens proteins, including developmental regulators, transcription factors, lens crystallins, cytoskeletal proteins, gap junction proteins and membrane channels [1, 2]. The best example of the latter is MIP, an aquaporin that not only facilitates the intercellular transport of water and small solutes [22], but also binds lens fiber cells together and ensures their optimal spacing, which is necessary for normal lens refraction behavior [26]. At least 19 mutations in the human *MIP* gene are associated with congenital cataracts, 11 of which are missense mutations, as well as two nonsense mutations, two frameshifts, two splice-site mutations, and one initiation codon mutation (Table 1). Here we identified the first *MIP* mutation associated with cataracts in the giant panda. It is a missense mutation in exon 4 (p.S229N) that replaces a highly-conserved serine residue with arginine in the intracellular C-terminal tail of the protein. This mutation was found in Jini (identified as S1 in Table 2) but not in 18 healthy controls representing all Jini’s traceable relatives as well as unrelated pandas from geographically diverse regions of China, supporting our hypothesis that p.S229N is a genuine disease-associated mutation and not an unrelated SNP. Jini’s father (S8) was sampled and did not carry the mutation, but no samples were available from Jini’s mother (who died in 2006) or Jini’s five offspring (two of whom have died, whereas one was exported to a foreign zoo). More distant relatives were also traced, including a female sibling of Jini’s parents who was also diagnosed with cataracts, but no samples were available. We also sampled the father (S11) and grandfather (S4) of Jini’s offspring and found no mutation. In the absence of informative pedigree-related samples, we acquired samples from pandas in Beijing, Baoxing, Ya’an, Wolong and Chengdu to ensure we captured broad genetic diversity.

Like other aquaporins, MIP features six transmembrane domains (H1–H6), three extracellular loops (A, C and E), and two intracellular loops (B and D), as well as intracellular N and C termini (Fig. 2) [18]. The C-terminal segment of the native protein is 44 amino acids in length (residues 220–263) and features an α-helix (residues 230–238) with an overlapping calmodulin-binding domain (residues 223–235) [55, 56] that regulates the permeability of the MIP water channels in response to Ca^2+^ [57, 58]. The C-terminal segment of MIP interacts not only with calmodulin, but also with the cytoskeletal protein filensin and the gap junction protein connexin 50 [59–61]. The novel mutation we identified lies within the calmodulin-binding domain at the N-terminal border of the α-helix, suggesting that the mutation may affect the permeability of MIP either constitutively or in response to Ca^2+^, or may disrupt its interaction with gap junctions and the cytoskeleton.

Several missense mutations associated with cataracts have been traced to exon 4 of the human *MIP* gene, but only one of these maps to the calmodulin-binding domain of the C-terminal segment, namely the R233K mutation identified by Lin et al. [31]. R233K is distal to our novel S229N mutation and lies within the α-helix as well as the calmodulin-binding domain, but like our mutation it replaces one residue with a chemically similar one, in this case the positively charged arginine to lysine, resulting in an autosomal dominant polymorphic binocular cataract. The S229N mutation in panda may have a similar effect, although we are unable to determine whether the cataract is polymorphic without other affected individuals (the Chinese family carrying the R233K mutation spanned six generations, with a wealth of clinical data). The presence of the cataract in Jini also suggests that the mutation is pathogenic and transmitted in an autosomal dominant manner, but both of Jini’s parents were apparently healthy and her father did not carry the mutation. We can only speculate that Jini represents a new germline mutation or that her mother was an unaffected carrier due to a lack of penetrance or expressivity, the latter being relatively common for congenital cataracts in human pedigrees [3].

Other mutations are known to truncate the C-terminal segment of MIP, which interferes with its trafficking to the plasma membrane and thus reduces or abolishes its activity [62]. The C-terminal regions spanning residues 223–234 and 235–263 are critical for protein transport from the cytoplasm to the plasma membrane [46, 63] and residue Ser235 is particularly important for MIP translocation to the plasma membrane following PKC-dependent phosphorylation [64]. Therefore, mutant versions of MIP lacking these residues become trapped in the cytoplasm, which restricts the formation of water channels in the plasma membrane and thus reduces lens fiber cell permeability and transparency. A long-terminal repeat inserted at the C-terminus of the mouse MIP protein was shown to disrupt lens fiber cell architecture in the *Cat*^*Fr*^ mutant, indicating that the C-terminal segment is also required for the development of the correct cellular architecture in the crystalline lens [47, 65, 66].

Part of the C-terminus is cleaved from MIP post-translationally such that mature lens fiber cells accumulate a truncated derivative (residues 1–246) rather than the full-length 263-residue protein. In transgenic knockout mice lacking a functional *MIP* gene, knocking in the C-terminal truncated sequence (making it the only version of MIP available throughout development) did not prevent the lens becoming opaque, and water permeability was reduced, but cell–cell adhesion was stronger than in the wild-type cells [67]. These results confirmed that full-length MIP is required for normal permeability although the truncated version does function as a water channel, and can be explained by the requirement of the complete C-terminal segment to traffic MIP to the plasma membrane. The truncation clearly plays an important role in cell–cell adhesion, which is enhanced when only the truncated MIP is available. The presence of our novel S229N mutation in this region of the panda *MIP* sequence indicates that the predicted structural alterations are likely to affect the structure and transparency of the lens by interfering with both permeability and cell–cell interactions. Our data provide more evidence of the pathogenic mechanisms of cataract formation in panda and extend the spectrum of known *MIP* gene mutations.

Clinically, the diagnostic criteria of age-related cataract are still controversial, and there is still no complete and accurate definition. In this study, the cataract occurrence of giant panda is associated with age, which belongs to the cumulative effect of pathogenic genes. Such pathogenic genes do not directly lead to the onset of early cataract as congenital cataract genes do. However, pathogenic genes accumulate harmful proteins or hinder the maintenance of lens function with the increase of age, eventually leading to cataract formation. This pathogenic gene like MIP gene mutation in this study might also be inherited to the offspring, and show senile cataract.

## Conclusions

We screened 11 genes often associated with human cataracts and identified a novel missense mutation (c.686G > A) in the *MIP* gene in a female panda diagnosed with progressive cortical punctate cataracts by using a functional candidate gene screening approach. This mutation was found in a captive giant panda with a unilateral cataract but not in 18 controls from diverse regions in China, suggesting it is most likely a genuine disease-associated mutation rather than a single-nucleotide polymorphism. The mutation could therefore serve as a new genetic marker to provide a new diagnostic tool for cataract risk assessment in captive giant pandas.

## Methods

### Proband and controls

Jini is a female giant panda who was born in 1993 in Beijing Zoo (China). Her mother was born in wild in 1981 and her father was born in Beijing Zoo in 1986. Both parents were healthy. Jini underwent examination at 28 years of age and was first diagnosed with senile cataract, but now also shows signs of corneal atrophy. She has poor vision and slow movement but no history of related systemic abnormalities. In addition to Jini (S1), we selected 18 healthy captive giant panda samples as controls, including Jini’s father (S8) and the father (S11) and grandfather (S4) of Jini’s offspring. The other samples (unrelated to Jini) were collected from pandas in Beijing, Baoxing, Ya’an, Wolong and Chengdu (Table 2).

### Mutation detection

We selected 11 candidate genes that are often associated with cataracts in humans (*CRYAB*, *CRYBA1*, *CRYBB1*, *CRYGC*, *HSPB6*, *HSPB7*, *HSPB9*, *GJA3*, *AQP3*, *MIP* and *HSF4*) and used them as functional candidates for mutation analysis in Jini and the controls. Peripheral venous blood samples (2 ml) were collected and stored at − 80 °C. Genomic DNA was extracted with phenol/chloroform (Sigma-Aldrich/Merck Millipore, Shanghai, China) immediately before analysis. Blood samples were collected in accordance with the Wildlife Protection Law of the People’s Republic of China (President of the People’s Republic of China No. 16), and the experimental approach was approved by the Beijing Zoo Academic and Ethics Committee.

PCR was carried out using the exon-spanning primers listed in **Table S****1**. Each 25-μl reaction comprised 1.5 mM MgCl_2_, 0.2 mM dNTPs, 0.5 μM of the appropriate forward and reverse primers, 2.5 U Taq DNA polymerase (TianGen, Beijing, China) and 20 ng genomic DNA in 1x PCR buffer (TianGen, Beijing, China). The samples were denatured at 95 °C for 5 min, followed by 34 cycles of 95 °C for 30 s, 57–63 °C (depending on the primer pair) for 30 s, and 72 °C for 30 s, and a final extension step at 72 °C for 10 min. The products were sequenced using an ABI 3730 Automated Sequencer (PE Biosystems, Foster City, CA, USA), analyzed using Chromas v2.33 and compared to the reference sequence in the NCBI database [68]. The sequence of the mutated giant panda MIP gene has been deposited in the NCBI GenBank database under accession number MT447398.

### Bioinformatics analysis

MIP amino acid sequences from five different species (human, bovine, rat, mouse and panda) were aligned and analyzed using CLC Free Workbench v4.5.1 (CLC Bio, Aarhus, Denmark). Protein hydrophilicity was determined using ProtScale [69]. The effects of the predicted amino acid substitution on the structure of MIP, and interactions between amino acid side chains, were predicted using SWISS-MODEL [70, 71], THREADER v3.5 [72, 73], Modeller v9.22 [74] and were visualized using Discovery Studio Visualizer. The damaging effects of the mutation were predicted using PROVEAN v1.1.3 (http://provean.jcvi.org/index.php).

## Supplementary Information

**Additional file 1.**

## Data Availability

Table S1 reports PCR primers designed for screening the candidate gene mutants. The sequencing data are publicly available at NCBI GenBank (accession number MT447398). All data necessary for confirming the conclusions of the article are present within the article, figures, and tables.

## Abbreviations

MIP: Major intrinsic protein
PITX3: Paired-like homeodomain 3
HSF4: Heat shock transcription factor-4
BFSP2: Beaded filament structural protein-2
GJA3: Connexin 46
GJA8: Connexin 50
AQP0: Aquaporin-0
AQP1: Aquaporin-1Quaporin-1
AQP3: Aquaporin-3Quaporin-3
SNPs: Single-nucleotide polymorphisms
CRYAB: Crystallin Alpha B
CRYBA1: Crystallin Beta A1
CRYBB1: Crystallin Beta B1
CRYGC: Crystallin Gamma C
HSPB6: Heat shock protein family B (Small) member 6
HSPB7: Heat shock protein family B (Small) member 7
HSPB9: Heat shock protein family B (Small) member 9
PKC-dependent phosphorylation: Protein kinase C dependent phosphorylation
