# Supplementary material for: A novel missense mutation in the gene encoding major intrinsic protein (MIP) in a Giant panda with unilateral cataract formation
Source: BMC Genomics. 2021 Feb 2;22:100. doi: 10.1186/s12864-021-07386-8 (PMC7856726; doi:10.1186/s12864-021-07386-8)
Supplement: Supplementary file 1 — Additional file 1. [file 12864_2021_7386_MOESM1_ESM.doc]

Table S1. PCR primers designed for screening the candidate genes.

| **Primer name** | **Forward (5′-3′)** | **Reverse (5′-3′)** |
| --- | --- | --- |
| CRYAB-1 | CCTGACTCCGTTCCAACTCC | CGGGCATCCTAATTAGAAGTTAGGGG |
| CRYAB-2 | GTCTGATGACCACATGGTAT | CCACTGGCTTAAAAATGGGA |
| CRYAB-3 | ATTTCAGATTTCTCAGACTCTCATT | AAGGACTAGAAAGGTTAAGTGAC |
| CRYBA1-1 | CTGAGATCAAGACCACAGCCTCCGA | ATTTCTTGGAGCCCCACGCAC |
| CRYBA1-2 | TGGGCGGCAGCTTACCTGCAT | TTCCACCCATCGCGGGCTTCC |
| CRYBA1-3 | CATCCCTAGCCTGGGCATGTA | GCCCCCAAAGCCAGTACCAAG |
| CRYBA1-4 | ATTACATTTACAAACCTAGTAACGT | CTCTTTTAGCTTAATGAAAATACCT |
| CRYBA1-5 | AAAGGCATTCTATTCTGTACCAATGGTG | TTCGACTCTTGGCTCAGGGCATG |
| CRYBB1-1 | GGACCGAGGGGTGGATTTGT | TCGCAGCCTTCTTTGAACTCATT |
| CRYBB1-2 | AGGCCTGGAGGAAGGCATTTCA | GTACCTCTAGGGAGGCTGGTGT |
| CRYBB1-3 | GCTGTGCAGGACTGCTTTTATTT | ATAATACCTCCCTCCTGAGCTTG |
| CRYBB1-4 | TAACTGGGGAAACATTTCGCTCACA | AAACCAGGGGCTCATGTTCTCATTT |
| CRYBB1-5 | ATTACTTCATTGAATCGTCATGACA | TTTAGAACCAATTATCCACGTTTGT |
| CRYBB1-6 | CCCCGTAATAAACCAGCCCTAGTGACC | AGCTGCCTTCCCCTGAGATCATG |
| CRYGC-1 | ACTGAATGCAGCACGTAAAAGGG | TGTGCATTTCTGTACCTGGGCTC |
| CRYGC-2 | AATGCAAACACGCTTTACATGCC | CCTCCCCACTTATTTGCTCCTCA |
| HSPB6-1 | CGCGCCTCAGCCCCGTTGCC | CCTCCTTCAGACTCCCCAACGCTCCC |
| HSPB6-2 | CAGGGGAAGGGGGTCTCATCACT | CCCGCCCCGTTCTGGCCCCAC |
| HSPB6-3 | CCAGAACGGGGCGGGGCATC | ACAGCTTTAGCACATTTATTGGGACAAC |
| HSPB7-1 | CTTGGCGTCTCGAATCCTGCTTGGTC | AGACAATACCGGAATCCCCTCTCTCA |
| HSPB7-2 | CCAGCCACACGCCAGCCCCTTTATGT | CTGGAGCCAAAGACTGGAGTTTGGGA |
| HSPB7-3 | GTGTGTGCGGGACTGTCAGCAGTG | CTGACCTCTGGGGCCACAACCGTT |
| HSPB7-4 | CACACAGTGTCACGCAGCCCACCT | TGGCTCCTAAAGAAAGGATCTCAGTGC |
| HSPB7-5 | AGCCCCTCTGCACCTGAGACTAGA | GCTGGCTTGGGGCTCAGGGAGTG |
| HSPB9 | ATGCAGCGGGTCGGTAGCGG | TCCATTATGCCTCAACCTCTGACCCCAACC |
| GJA3-1 | GCTGCGGACGTTTATCTAA | GGGGAGTCTGAGCGATAGT |
| GJA3-2 | CTTCCTGTACGGCTTTGAG | TGGGTTGTTGTGAATACCT |
| GJA3-3 | TCCAGACAGCTTTATAATA | AACAAACAGCGACTCAGAT |
| AQP3-1 | CACCATTGCCTCTCAGACCCG | GAGCTGTCAGTGGGAGTAACT |
| AQP3-2 | AAATTAAAGTTTGGGCATTGT | TGATAATCGGATGCCAAGGTG |
| AQP3-3 | AGAAACTTGACACTTTGAACC | ACGTGACTGCTGTATTACTCA |
| AQP3-4 | GCTGCCCACCCTCCAAAGACT | TTCCCGTGTTCAGCCCTTTCC |
| AQP3-5 | CTTAACAAAACAGGAATTGGC | CTTCACATTCTCCTGCTCGTT |
| AQP3-6-1 | TCCCCGCCTTTTCACCGCCAT | AGACCCCTCGCACTTTCCTCA |
| AQP3-6-2 | ACGAGCAGTGGGTGTGTGAGC | GTGGGGCCGCCGAGTTCAAGC |
| MIP-1 | CTCTGCTTCTCTCCCAGT | GAACAAAAGCTGAAACCA |
| MIP-2 | TGGTTTCAGCTTTTGTTC | AGACAAACTGGATAGGGG |
| MIP-3 | TATTCCTCTCTTCTGTGA | ATAATGTGCTAGAACTGG |
| MIP-4 | ACACTGGTTTCTGCTTAA | AACACACACATAAATTAA |
| HSF4-1 | GCGTACGGGTGATAACTCC | CCATCTCACACACCCCCTC |
| HSF4-2 | CTTGTCCCATGTCTCCAGG | CAGAATGGTAGGTCTAAAC |
| HSF4-5-6 | GACCGCTCCTTCCTCTCTCCT | ATTGGGGGGAGAGGGAAAGTC |
| HSF4-7 | CCCTGTGTTCCCAAATTTTCC | TAAGGAGTAAGGTTTGAGGGG |
| HSF4-8-9 | TCCTTTCCCCCATGTCCCTAA | TTGACCTGATGGGAGTTGCTA |
| HSF4-10-12 | ATCCCAAGACCCCAGTTCCAT | ATCCATTCTCAACCTGCCCAC |
| HSF4-13 | TTAAGCTACCCATCTTCTCGA | TGAACCCTCTCTGCTTATCCG |
